# Self-induced redox cycling coupled luminescence on nanopore recessed disk-multiscale bipolar electrodes†
†Electronic supplementary information (ESI) available. See DOI: 10.1039/c5sc00433k



**DOI:** 10.1039/c5sc00433k

**Published:** 2015-03-25

**Authors:** Chaoxiong Ma, Lawrence P. Zaino III, Paul W. Bohn

**Affiliations:** a Department of Chemistry and Biochemistry , University of Notre Dame , Notre Dame , IN 46556 , USA . Email: pbohn@nd.edu; b Department of Chemical and Biomolecular Engineering , University of Notre Dame , Notre Dame , IN 46556 , USA

## Abstract

We present a new configuration for coupling fluorescence microscopy and voltammetry using self-induced redox cycling for ultrasensitive electrochemical measurements. An array of nanopores, each supporting a recessed disk electrode separated by 100 nm in depth from a planar multiscale bipolar top electrode, was fabricated using multilayer deposition, nanosphere lithography, and reactive-ion etching. Self-induced redox cycling was induced on the disk electrode producing ∼30× current amplification, which was independently confirmed by measuring induced electrogenerated chemiluminescence from Ru(bpy)_3_^2/3+^/tri-*n*-propylamine on the floating bipolar electrode. In this design, redox cycling occurs between the recessed disk and the top planar portion of a macroscopic thin film bipolar electrode in each nanopore. Electron transfer also occurs on a remote (mm-distance) portion of the planar bipolar electrode to maintain electroneutrality. This couples the electrochemical reactions of the target redox pair in the nanopore array with a reporter, such as a potential-switchable fluorescent indicator, in the cell at the distal end of the bipolar electrode. Oxidation or reduction of reversible analytes on the disk electrodes were accompanied by reduction or oxidation, respectively, on the nanopore portion of the bipolar electrode and then monitored by the accompanying oxidation of dihydroresorufin or reduction of resorufin at the remote end of the bipolar electrode, respectively. In both cases, changes in fluorescence intensity were triggered by the reaction of the target couple on the disk electrode, while recovery was largely governed by diffusion of the fluorescent indicator. Reduction of 1 nM of Ru(NH_3_)_6_^3+^ on the nanoelectrode array was detected by monitoring the fluorescence intensity of resorufin, demonstrating high sensitivity fluorescence-mediated electrochemical sensing coupled to self-induced redox cycling.

## Abstract 2

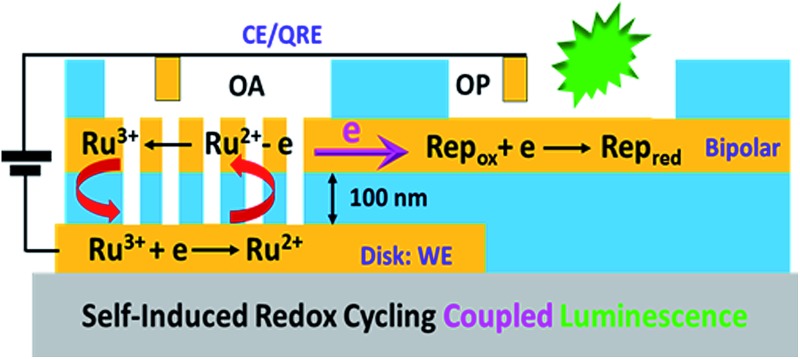
Self-induced redox cycling at nanopore ring-disk electrodes is coupled, through a bipolar electrode, to a remote fluorigenic reporter reaction.

## Introduction

The combination of fluorescence spectroscopy with electrochemistry presents new avenues for the study of redox reaction events, with potential for enhanced throughput, sensitivity, and spatial resolution.^1–6^ For example, complementary optical and electrochemical information provided by coupling amperometry and total internal refection fluorescence microscopy has been employed to unravel the mechanism of exocytosis events on single cells.^7,8^ Fluorescence-detected cyclic voltammetry has also been used to study thermodynamic and kinetic characteristics of electron transfer in immobilized proteins, exhibiting variation in fluorescence due to surface heterogeneity.^6,9^ The coupling of electron transfer events to fluorescence allows the inherently low background of fluorescence measurements to be exploited, as shown by the demonstration of single molecule sensitivity in monitoring charge-transfer events in reversible redox dyes that switch between fluorescent and nonfluorescent states upon potential modulation.^10–13^

Nonfluorescent redox couples may also be studied by monitoring the interaction between the couple and a fluorescent dye. For example, pH-sensitive fluorescent indicators have been used to monitor electrochemically generated H^+^.^10,14^ This simple, but effective, strategy has been developed into a combinatorial method for simultaneous parallel screening of electrocatalysts for the oxidation of methanol.^5^ Alternatively, electrochemical reactions of nonfluorescent analytes can be coupled to electrogenerated chemiluminescence (ECL) using a bipolar electrode which acts as anode and cathode simultaneously.^15,16^ This configuration allows the redox reaction of the target couple to be monitored through ECL intensity changes at the indicator.^17^ Zhang and coworkers have exploited this type of coupling reaction using bipolar electrodes^18,19^ to develop fluorescence-enabled electrochemical microscopy, which can monitor a large number of parallel redox events by fluorescence imaging.^2^

In an effort to improve the sensitivity of electrochemical detection, dual electrodes with μm- to nm-scale spacing have been fabricated to take advantage of redox cycling (RC) to amplify redox events and enhance the measured current.^20–25^ The RC effect, relying on the cycling of the redox species between two closely spaced electrodes, can provide up to 1000-fold current amplification, achieving single molecule detection in favorable circumstances.^23–26^ In addition, RC electrodes can be integrated within microfluidic systems to execute hydrodynamic voltammetry thereby avoiding the loss of sensitivity due to transport across a diffusive boundary layer.^22,26–28^ The RC effect can also improve selectivity to species exhibiting different degrees of reversibility, as well as different redox potentials.^22,27,29,30^ In addition, RC is compatible with microfluidic systems, thus holding promise for lab-on-a-chip devices.^20,22,24,27,28^ Therefore, it is reasonable to ask whether coupling of RC to fluorescence detection could combine the advantages of the individual techniques to achieve singular sensitivity and selectivity of in the study of redox reactions.

In a typical RC measurement, two electrodes are held at potentials negative and positive of the analyte redox potential to initiate and sustain electrochemical cycling.^21,24,26^ Surprisingly, self-induced redox cycling (SIRC) can be observed when a powered electrode is placed adjacent to a nearby unbiased (floating) electrode.^31–34^ In this situation, depletion of redox species at the working electrode produces a location-dependent concentration polarization relative to the adjacent electrode. These local concentration differences can be sufficient to drive oxidation and reduction reactions occurring on the unbiased electrode.^31,34^ This constitutes a bipolar electrode so that electrons flowing to the other end can balance the charge induced by the localized redox reaction, as has been previously monitored using simple voltage measurements and confirmed by stripping voltammetry.^30–32,35,36^

**Fig. 1:**
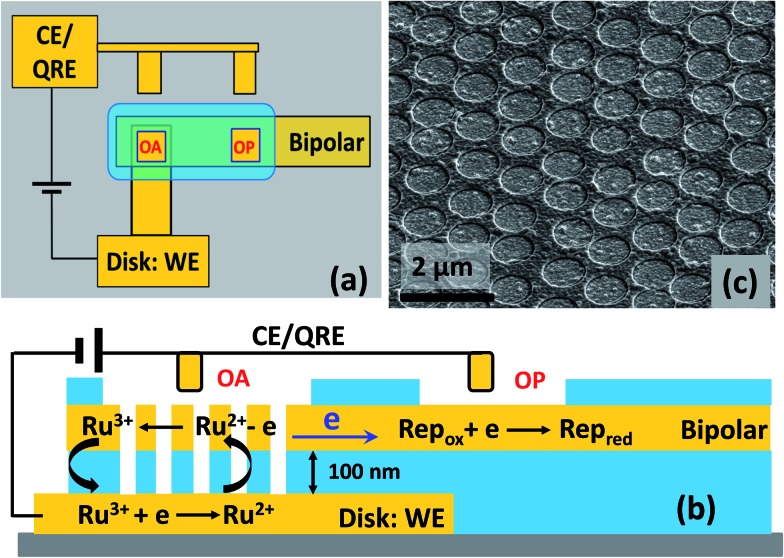
(a) Schematic diagram showing a top view of the macroscopic layout of the BRDE array and remote electrode separated by mm-distances; (b) schematic side view of the BRDE array and illustration of the mechanism of self-induced redox cycling for Ru(NH_3_)_6_^2/3+^ (abbreviated as Ru^2/3+^). SIRC in the BRDE at OA is monitored by a reporter redox couple (Rep_ox/red_) at a remote location (OP); (c) SEM image of the array at 45° tilt.

In the present study, the SIRC effect is employed to couple redox reactions of a target redox couple, at a nanopore-confined recessed disk electrode, with inherently low background fluorescence measurements at the remote end of a multiscale bipolar electrode. The exposed bipolar electrode is multiscale, because the nanopore portion is separated from the recessed disk electrode by 100 nm, while the remote end of the electrode is in contact with a different solution located ∼1 mm away, *viz.* Fig. 1. The SIRC effect is studied by measuring the cyclic voltammetry (CV) of a model redox couple, Ru(NH_3_)_6_^2/3+^, using the recessed disk electrodes as working electrodes (WE) and the nanopore portion of the bipolar electrode as the collector in a generator–collector arrangement. In order to confirm the participation of the floating nanopore bipolar electrode in SIRC, electrogenerated chemiluminescence (ECL)^17,37–39^ from Ru(bpy)_3_^2+^ and tri-*n*-propylamine, was monitored at the remote end of the multiscale bipolar electrode. To accomplish SIRC-coupled fluorescence, the multiscale bipolar electrode was placed in contact with a redox-switchable fluorescent species in the remote cell. SIRC in the nanopore bipolar recessed disk electrode (BRDE) array was detected by the oxidation of non-fluorescent dihydroresorufin to fluorescent resorufin and corresponding increase in fluorescence in the remote cell.^2,40^ Increasing fluorescence due to electrochemical oxidation of non-fluorescent dihydroresorufin to fluorescent resorufin indicates an oxidation reaction at the recessed disk WE, while reduction of resorufin to dihydroresorufin and decrease of fluorescence indicates reduction at the WE.^18^ This scheme was validated using Ru(NH_3_)_6_^3+^, demonstrating sensitivity to concentrations as low as 1.0 nM Ru(NH_3_)_6_^3+^.

## Experimental section

### Reagents and materials

All chemicals, including hexaammineruthenium(iii) chloride (Ru(NH_3_)_6_Cl_3_), tris(2,2′-bipyridyl) dichlororuthenium(ii) hexahydrate (Ru(bpy)_3_Cl_2_), tri-*n*-propylamine, resorufin, ferrocenemethanol, glucose, and underivatized polystyrene spheres (1.0 μm in diameter, 10 wt%), were obtained from Sigma-Aldrich and used as received. All solutions for electrochemical measurements were prepared from deionized (DI) water (*ρ* ∼ 18 MΩ cm) generated by a Milli-Q Gradient water purification system (Millipore).

### Device fabrication

Similar to the procedure used for developing recessed ring-disk electrode arrays,^20,22,26^ multiscale BRDE arrays were fabricated *via* layer-by-layer deposition, nanosphere lithography, and a reactive ion etching (RIE) process. Photolithography was used to define the bottom Au electrode and the etched areas on both the array and planar electrodes (see Fig. S1, ESI†). The thickness of the bottom Au, middle silicon nitride (SiN_*x*_), top Au, and top SiN_*x*_ layers are 200 nm, 100 nm, 50 nm, and 200 nm, respectively. Fig. 1 shows a schematic diagram and an SEM image of a fabricated array containing cylindrical nanopores of ∼700 nm diameter with ∼1.0 μm pitch. The size of the first open area (OA) above the BRDE array is 100 μm × 100 μm. The second open area (OP) on the remote end of the bipolar electrode, varying with size, is 500 μm or 6 mm away from OA.

### Electrochemical and fluorescence measurements

Electrochemical experiments were conducted on a CHI bipotentiostat (842c, CH Instruments Inc.) using a Ag/AgCl reference electrode or thin film Au quasi-reference electrode (CE/QRE, Fig. 1). Tri-*n*-propylamine (TPA) (1.0 M) was dissolved in 1.0 M HCl prior to mixing with Ru(bpy)_3_Cl_2_ aqueous solution. The solutions of analytes and resorufin were prepared in 0.2 M phosphate buffer (pH 7) and purged with N_2_ for ≥5 min prior to use. Dihydroresorufin (0.1 mM) was obtained by mixing 0.1 mM resorufin with 0.5 M NaOH and 50 mM glucose.^41^ For all electrochemical and ECL measurements, ∼30 μL solution was added to a PDMS well covering the electrodes. In the fluorescence measurements, analyte of Ru(NH_3_)_6_^3+^ or ferrocenemethanol was added to a PDMS well covering the OA portion, while 2 μL of resorufin or dihydroresorufin solution was added to the remote electrode area (OP, Fig. 1) and covered by a coverslip. Chronoamperometry was performed by applying potential steps for 1 s, followed by a return to a rest potential for a 5 s recovery period between steps. Fluorescence and ECL measurements were performed on an epifluorescence microscope (IX71, Olympus) equipped with an X-Cite 120 PC illumination system (Exfo) and a TRITC filter set (Chroma). All images were acquired using a 10×, 0.25 NA lens and recorded at 10 frames per second using an electron-multiplier CCD camera (PhotonMax512, Princeton Instruments). WinView (Princeton Instruments) and ImageJ (NIH) software packages were used for acquiring images and data analysis, respectively.

## Results and discussion

### Self-induced redox cycling on the BRDE array

**Fig. 2:**
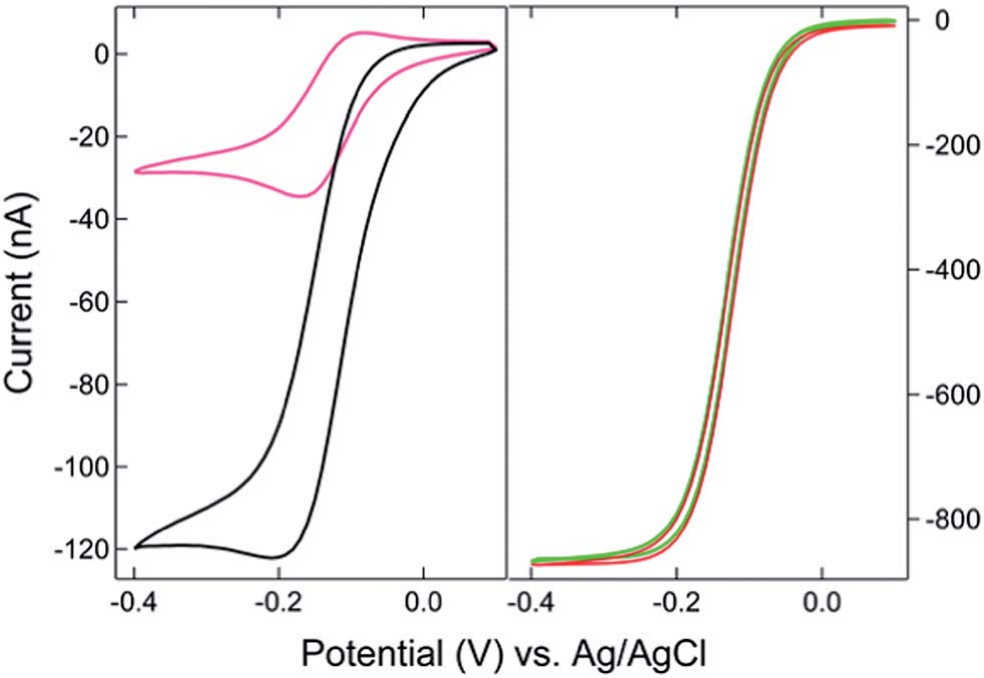
(*Left*) Cyclic voltammograms of 1 mM Ru(NH_3_)_6_^3+^ on a BRDE array with only OA contacting solution (black) and on a recessed microdisk electrode array of the same size (pink). (*Right*) CVs on the array with both OA and OP (1 mm × 5 mm) in contact with 1 mM Ru(NH_3_)_6_^3+^. The bipolar electrode is floating (red) or held at +0.1 V (green), respectively, *vs.* Ag/AgCl.

The SIRC effect has been observed previously on interdigitated electrode arrays and planar-recessed disk electrode arrays with μm-scale spacing.^31,34^ In order to increase the efficiency of redox cycling and generate larger current amplification, multiscale BRDE arrays, with a nanopore interelectrode spacing of 100 nm, were fabricated in this study. Fig. 1 illustrates the layout of the BRDE array prepared with two openings – one on the BRDE array (OA) and another on the remote portion of the bipolar electrode (OP). Fig. 2 shows the results of CV measurements of 1 mM Ru(NH_3_)_6_^3+^ on the BRDE array. In order to reveal the effect of the bipolar electrode in redox cycling, voltammograms obtained on a recessed microdisk electrode array of the same size, *i.e.* with the same geometry as the BRDE array but without a top Au layer, are used for comparison.

With the planar electrode floating and only OA in contact with the solution, a pseudo-steady-state response with limiting current of 120 nA was observed, a value 4-fold larger than the peak current (30 nA) obtained on the recessed microdisk electrode array. This change in CV response and increase in faradaic current are attributed to the SIRC effect mediated by the redox reactions occurring on the nanopore portion of the floating bipolar electrode. The SIRC effect is more pronounced when the remote portion of the bipolar electrode (OP) is exposed to Ru(NH_3_)_6_^3+^ solution, *i.e.* Ru(NH_3_)_6_^2/3+^ is the reporter species. Indeed, the resulting current amplification increases with the area of OP on the planar electrode that contacts the solution (Fig. S2, ESI†). With a sufficiently large OP area (1 mm × 5 mm), a steady-state response (Fig. 2, red curve) with maximized SIRC effect is achieved with SIRC current equivalent to that obtained by biased RC with the collector electrode at +0.1 V *vs.* Ag/AgCl (Fig. 2, green curve). The limiting current obtained on the BRDE array under these conditions is ∼870 nA, yielding an amplification factor of ∼30 and confirming the participation of the floating bipolar electrode in redox cycling.

The proposed mechanism for SIRC on the BRDE array is illustrated by Fig. 1(b), which is similar to those suggested previously for interdigitated electrode and microscale planar recessed-disk electrode arrays.^31,34^ The reduction of Ru(NH_3_)_6_^3+^ to Ru(NH_3_)_6_^2+^ on the disk electrode produces a location-dependent concentration polarization of Ru(NH_3_)_6_^3/2+^ at the nanopore bipolar electrode. Specifically, with a reducing potential applied at the disk electrode, the ratio of Ru(NH_3_)_6_^3+^ to Ru(NH_3_)_6_^2+^ is much larger at OP than at OA. As a result, reduction of Ru(NH_3_)_6_^3+^ at OP is coupled to oxidation of Ru(NH_3_)_6_^2+^ at OA, Fig. 1(b), in order to maintain electroneutrality of the bipolar electrode. This then facilitates SIRC on the BRDE array.

### SIRC effect observed by ECL

**Fig. 3:**
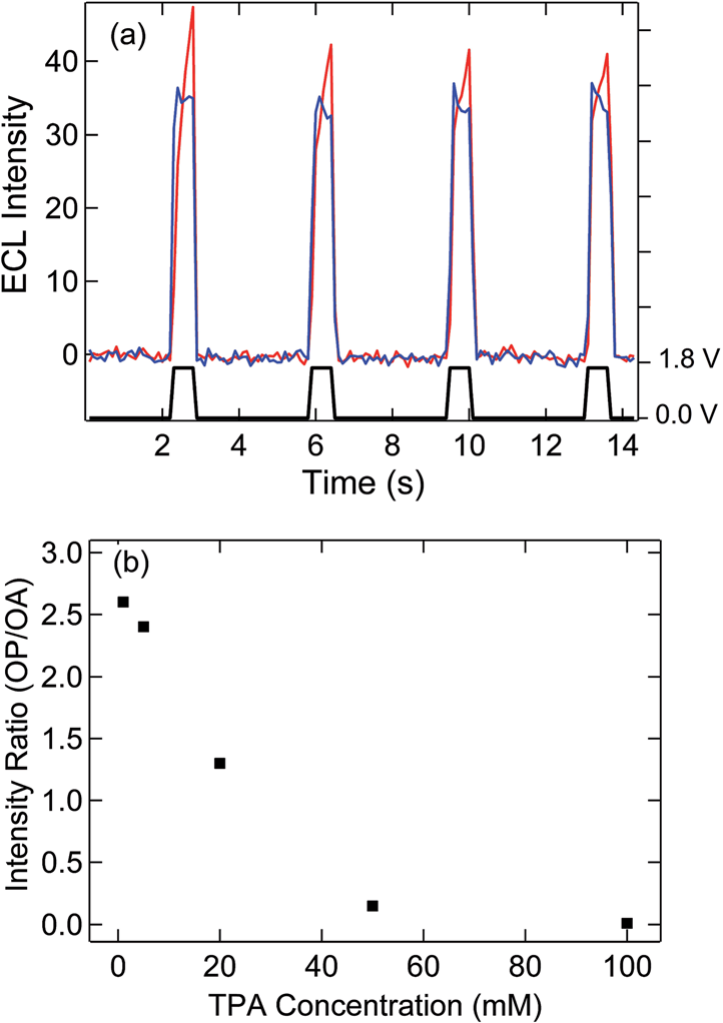
(a) ECL measurement in 5 mM Ru(bpy)_3_^2+^ and 5 mM TPA solutions on the multiscale bipolar electrode at OA (blue) and OP (red) in the same PDMS well. Multiple potential steps switching between 1.8 V and 0 V *vs.* QRE (black) were applied on the disk with the bipolar electrode floating. (b) Ratio of ECL intensities at OP and OA as a function of TPA concentration.

Electron transfer at a floating electrode with SIRC has been previously observed on interdigitated electrode arrays.^31,32,34,36^ In the present study, ECL was used to monitor the induced redox reaction at the unbiased bipolar electrode with OP located 500 μm away from OA. Potential steps were applied to the disk electrode with the bipolar electrode floating and both electrodes in contact with 5 mM Ru(bpy)_3_^2+^ and varying concentrations of TPA. As shown in Fig. 3(a), potentially modulated ECL was observed on OA due to oxidation and subsequent reaction of Ru(bpy)_3_^2+^ with TPA, thereby generating chemiluminescence.^37–39^

Similar to the mechanism given in Fig. 1, the oxidation of Ru(bpy)_3_^2+^ to Ru(bpy)_3_^3+^ at recessed disk electrode at OA leads to a gradient in the concentration of Ru(bpy)_3_^3/2+^ between the exposed portions of the bipolar electrode at OP and OA. Accordingly, reduction of Ru(bpy)_3_^3+^ and oxidation of Ru(bpy)_3_^2+^ occur on the OA and OP areas of the bipolar electrode, respectively, to counteract this difference and maintain electroneutrality. ECL with a similar potential dependence to that observed at OA, Fig. 3(a) is observed at OP, thus confirming the participation of the floating bipolar electrode in redox cycling. The slight delay of the self-induced ECL response is assigned to the accumulation of Ru(bpy)_3_^3+^ at OA, which is required to trigger the oxidation of Ru(bpy)_3_^2+^ and TPA at OP, since TPA is oxidized at a more positive potential. The induced ECL intensity at OP is significantly affected by the concentration of TPA, Fig. 3(b) and Fig. S3,† which decreases the availability of Ru(bpy)_3_^3+^ at OA and the resulting concentration difference that governs the ECL at OP. In solutions with a 10 : 1 or higher ratio of TPA : Ru(bpy)_3_^2+^, ECL at OP is almost entirely eliminated. This observation is attributed to consumption of Ru(bpy)_3_^3+^ produced on the disk by the TPA,^39^ thereby disrupting the concentration difference across OA and OP portions of the bipolar electrode. The significant decrease of ECL intensity at high concentration of TPA was not observed on the nanopore array (OA) or on a microelectrode under similar ECL measurement conditions (Fig. S4†), the latter exhibiting relatively strong, TPA concentration-independent emission above 50 mM TPA. These results indicate that a concentration difference across the two ends of the bipolar electrode is crucial for induced reactions on the OP portion of the bipolar electrode and SIRC at the nanopore electrode array (OA).

### SIRC coupling with fluorescence microscopy

Electron transfer at the floating bipolar electrode, confirmed by the above ECL results, can be employed to couple SIRC to fluorescence emission, such that a redox reaction at the nanopore-recessed disk electrode (OA) can be monitored by fluorescence at the OP portion of the multiscale bipolar electrode. Separation of the target redox couple from the reporter is invaluable, for example, in eliminating potential interferences in analytical measurements. To test this hypothesis, the SIRC effect was first measured by contacting OA and OP with two different solutions. OA was filled with 1 mM Ru(NH_3_)_6_^3+^, while OP was filled with Ru(NH_3_)_6_^3+^ of different concentrations. With the introduction of the target solution to OP, an obvious increase of faradic current can be seen at the disk electrode at OA (Fig. S5†). With a fixed OP area (100 μm × 100 μm), larger current amplifications are obtained on the array with higher Ru(NH_3_)_6_^3+^ concentrations at OP, since these produce larger concentration differences between OP and OA.

**Fig. 4:**
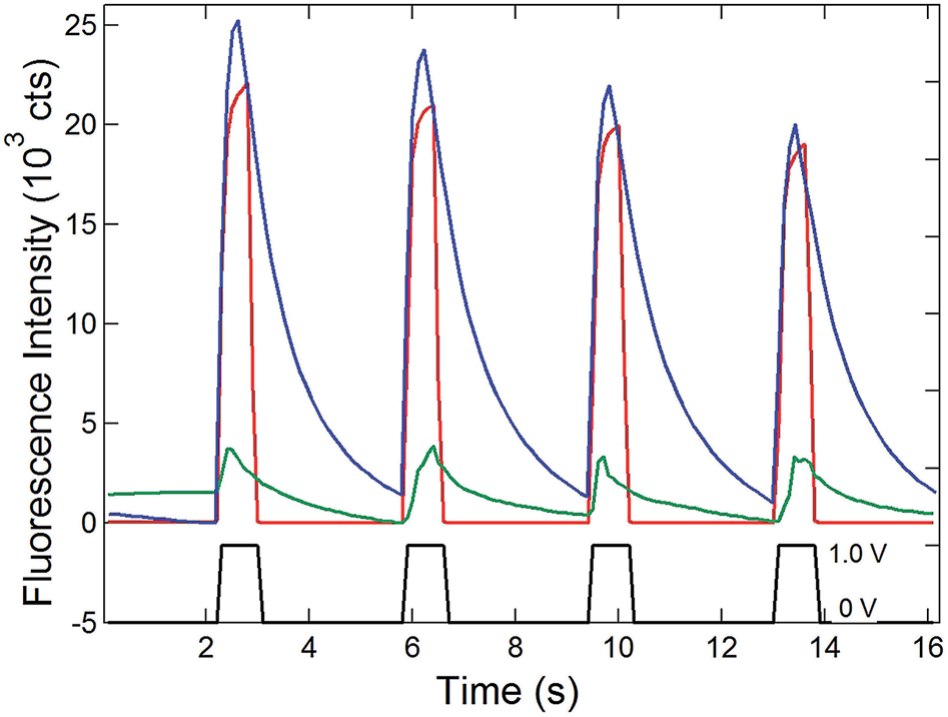
Comparison of electrochemically-induced fluorescence and SIRC-coupled fluorescence in solutions of 0.1 mM dihydroresorufin at the OP portion of the bipolar electrode. Potential steps between 0 V and 1.0 V *vs.* QRE (black) were applied at the recessed disk electrode array (OA) filled with 100 μM (blue) and 1 μM (green) ferrocenemethanol solutions in a PDMS well. Red curve: potential steps applied directly to the bipolar electrode at OP with no solution in the OA portion of the bipolar electrode (intensity × 0.1).

A similar structure was then used to investigate the coupling of fluorescence to voltammetry. The exposed spot OP was filled with 0.1 mM dihydroresorufin, a nonfluorescent phenoxazine dye that can be oxidized to resorufin, the latter exhibiting strong fluorescence.^41,42^ The potential modulated change of fluorescence intensity of dihydroresorufin has been observed previously^41^ and was confirmed here by direct electrochemical oxidation, Fig. 4 (red curve). Similar switching of fluorescence was observed on the floating bipolar electrode at OP when potential steps were applied to the disk electrode sufficient to oxidize ferrocenemethanol (Fc) to ferriceniumethanol (Fc^+^). This observation is consistent with the SIRC effects reported above; Fc^+^ generated at the recessed nanopore disk WE is reduced to Fc at the OA portion of the bipolar electrode, which is then coupled to the oxidation of dihydroresorufin (H_2_RF) to resorufin (RF) at OP. The overall reaction,

1 Fc^+^ + H_2_RF ↔ Fc + RF

should occur spontaneously, since Fc^+^/Fc has a more positive standard reduction potential (∼0.2 V *vs.* Ag/AgCl)^18,43^ than the resorufin couple (∼–0.1 V *vs.* Ag/AgCl).^42^ The degree of reaction and the resulting fluorescence intensity should therefore depend on the concentration of Fc, which is confirmed by comparison of the 100 μM and 1 μM results in Fig. 4. It is interesting to note that the recovery of the fluorescence to baseline is slower in the presence of SIRC than with direct potential-modulation. This observation is reasonable, since no reaction occurs at the recessed disk WE at 0 V in the presence of Fc. Instead, the recovery from fluorescent state to nonfluorescent state (baseline) relies on the diffusion of H_2_RF from the bulk solution to replace the RF on the electrode surface at OP, which is a slow process compared to the potential-driven reaction.

**Fig. 5:**
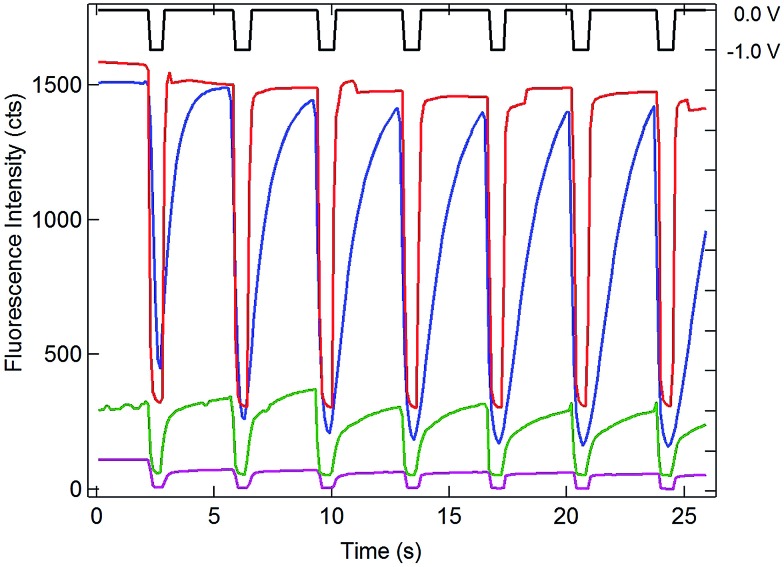
Comparison of electrochemically-induced fluorescence and SIRC-coupled fluorescence in solutions of 0.1 mM resorufin at the OP portion of the bipolar electrode. Potential steps between 0 V and –1.0 V *vs.* QRE (black) applied at the recessed disk WE at OA for 1 mM (blue), 1 μM (green), and 1 nM (purple) Ru(NH_3_)_6_^3+^ in a PDMS well. Red curve: potential steps applied directly to the bipolar electrode at OP with no solution covering the OA portion of the bipolar electrode (Intensity displayed at 0.03×). Offsets used to enhance visualization.

Having established the efficient coupling of redox reactions in the nanopore array at OA to fluorigenic reactions at the remote end of the bipolar electrode, the approach was used to monitor the presence of analyte at OA, using the H_2_RF/RF reporter system at OP. The results for determination of Ru(NH_3_)_6_^3+^ at concentrations in the range 1 mM to 1 nM are given in Fig. 5. Applying a reducing potential to the recessed disk WE results in decreased fluorescence, similar to the potential-driven behavior observed with the Fc/Fc^+^ couple. The overall coupling reaction is,

2 RF + Ru(NH_3_)_6_^2+^ ↔ H_2_RF + Ru(NH_3_)_6_^3+^

Similar to Reaction (1), this reaction occurs spontaneously, since the standard reduction potential of H_2_RF/RF is more positive than that of Ru(NH_3_)_6_^2/3+^ (–0.2 V *vs.* Ag/AgCl).^22,34^ Also similar to the behavior of the Fc/Fc^+^ couple at OA, recovery of the fluorescence baseline intensity at 0 V is a slow, diffusion-controlled process. Again, this is reasonable, since reduction of Ru(NH_3_)_6_^3+^ at the WE at 0 V is not spontaneous.

Since one goal of this study was to exploit the coupling of fluorescence emission and electrochemistry to improve the sensitivity of redox cycling measurements, Ru(NH_3_)_6_^3+^ determinations were also performed at lower concentrations using the same electrode geometry. Similar behavior with detectable change in fluorescence intensity could be observed down to 1 nM Ru(NH_3_)_6_^3+^ (purple curve, Fig. 5). This excellent sensitivity is attributed to the combination of redox cycling, that amplifies the redox event, and the inherently low background of fluorescence measurements. Given the direction of the fluorigenic reaction, reductions at the WE are accompanied by a high-to-low fluorescence transition, as seen in Fig. 5, which is not ideal from a measurement perspective. Thus, although a complete delineation of the analytical figures-of-merit is beyond the scope of the present work, limits of detection below 1 nM for this model analyte are clearly achievable by SIRC-coupled fluorescence measurements. Furthermore, oxidation reactions at WE, which are signified by increases in fluorescence emission (*cf.* Fig. 4), should provide even better performance.

## Conclusion

These experiments introduce a unique multiscale bipolar electrode scheme to couple self-induced redox cycling in nanopore arrays to electrochemistry and fluorescence of a reporter redox pair at a location remote from the target redox couple. The nanopore end of the bipolar electrode is placed adjacent (100 nm) to a recessed disk electrode, allowing efficient transport between the two electrodes in order to achieve redox cycling. In contrast, the remote end of the bipolar electrode is placed in a cell far from the BRDE to implement highly efficient reporter measurements, *e.g.* using redox-coupled fluorigenic reactions. Together these characteristics permit the effective coupling of redox cycling with fluorescence measurements for ultrasensitive electrochemical detection. SIRC with current amplification up to 30-fold was observed on the BRDE array with an unbiased planar electrode in CV measurements. Although not as large amplification factors achievable with biased collector electrodes, the participation of the bipolar electrode in redox cycling and its electrical connection to the remote end of the electrode permit the target event and the reporter reaction to spatially separated, greatly reducing measurement background. ECL measurements using Ru(bpy)_3_^2+^/TPA were used to elucidate the role of concentration polarization at both ends of the bipolar electrode. At the nanopore BRDE array, concentration polarization is engendered directly, using the recessed disk WE to manipulate the concentration of one member of a redox couple to induce the reverse reaction at the nanopore-bipolar electrode. The effects of concentration polarization at the remote end of the bipolar electrode were demonstrated through the effect that changing TPA concentration has on ECL emission intensities. Finally, the multiscale bipolar electrode efficiently couples SIRC amplification to fluorescence readout in electrochemical determinations, as shown by the coupling of a model redox couple, Ru(NH_3_)_6_^2/3+^ in the BRDE array to fluorescence detection using an H_2_RF/RF reporter system at the remote end. Oxidation of ferrocenemethanol and reduction of Ru(NH_3_)_6_^3+^ were both optically monitored using changes in the fluorescence intensity reflecting local concentrations of RF at the remote location. Ru(NH_3_)_6_^3+^ was determined at concentrations as low as 1 nM, indicating significant promise for ultrasensitive detection.
