# Supplementary material for: Self-induced redox cycling coupled luminescence on nanopore recessed disk-multiscale bipolar electrodes
Source: Chem Sci. 2015 Mar 25;6(5):3173–9. doi: 10.1039/c5sc00433k (PMC5490416; doi:10.1039/c5sc00433k)
Supplement: Supplementary file 1 [file SC-006-C5SC00433K-s001.pdf]

Electronic Supplementary Material (ESI) for Chemical Science.
This journal is © The Royal Society of Chemistry 2015

Supplemental Information for

Self-Induced Redox Cycling Coupled Luminescence on Nanopore Recessed Disk-Multiscale Bipolar Electrodes

Chaoxiong Ma[1], Lawrence P. Zaino III[1], and Paul W. Bohn[1,2*]

[1]Department of Chemistry and Biochemistry, University of Notre Dame, Notre Dame, IN 46556

[2]Department of Chemical and Biomolecular Engineering, University of Notre Dame, Notre Dame, IN 46556

1. Fabrication of Bipolar-Recessed Disk Electrode (BRDE) Nanoarrays

Using procedures similar to those developed previously to fabricate recessed ring-disk electrode arrays,[1] BRDE arrays were fabricated on glass slides using layer-by-layer deposition, nanosphere lithography, and a reactive ion etching (RIE) process (Fig. S1)

a) After cleaning with piranha solution, the glass slides were patterned by photolithography using AZ5214E photoresist (PR) to define an electrode area of 200 μm x 200 μm (WE in layout). A 200-nm thick Au film, using 5 nm Cr as an adhesion layer, was deposited by thermal evaporation. The undeveloped photoresist was then removed by rinsing with acetone.

b) A 100 nm silicon nitride (SiN_x) layer was then deposited on the Au electrode by plasma-enhanced chemical vapor deposition.

c) The substrates were then coated with a compact monolayer of polystyrene (PS) spheres to template the electrode array using a nanosphere lithography approach reported previously.[2] Briefly, 5.0 wt% PS solution (in 1:1 methanol/water) was spread onto the surface of water in a petri dish, producing a monolayer of PS spheres at the air-water interface. This PS

monolayer was transferred to the electrode surface by partially dipping the wetted substrate into the water. The PS-decorated substrate was then exposed to an O_2 plasma (50 W) for 8 min to reduce the size of the spheres to *ca*. 700 nm.

d) Using an Al mask, the top Au (50 nm) electrodes (Planar and CE/QRE in layout) were deposited to overlap the bottom electrode covered by SiNx film and PS spheres.

e) The PS spheres were removed by ultrasonicating the substrates in $CHCl_3$ for 5 min, followed by a 10 min exposure to a strong (1500 W) O_2 plasma was used to remove the PS spheres.

f) An additional 200 nm of SiN_x was deposited over the entire substrate after removal of the PS.

g) Photolithography was then used to develop the areas of interest to be selectively exposed to the following etching process. The first area (OA) of dimensions 100 μm x 100 μm was opened over the nanopore BRDE array, and a second area (OP) with size varying from 100 μm x 100 μm to 1 mm x 5 mm was opened over the remote portion of the bipolar electrode. The distance between OA and OP was either 0.5 mm or 6 mm. The contact pads of the electrodes were defined by using PR as an etch mask (not shown in Fig S1).

h) The SiN_x layer on the OA and OP contact pads was removed by reactive ion etching using a mixture of CF_4 (45 sccm) and O_2 (5 sccm) at 50 mTorr and 50 W rf power for 3 min. Acetone cleaning followed by exposure to a high power O_2 plasma (1500 W, 10 min) were used to remove the PR after etching.

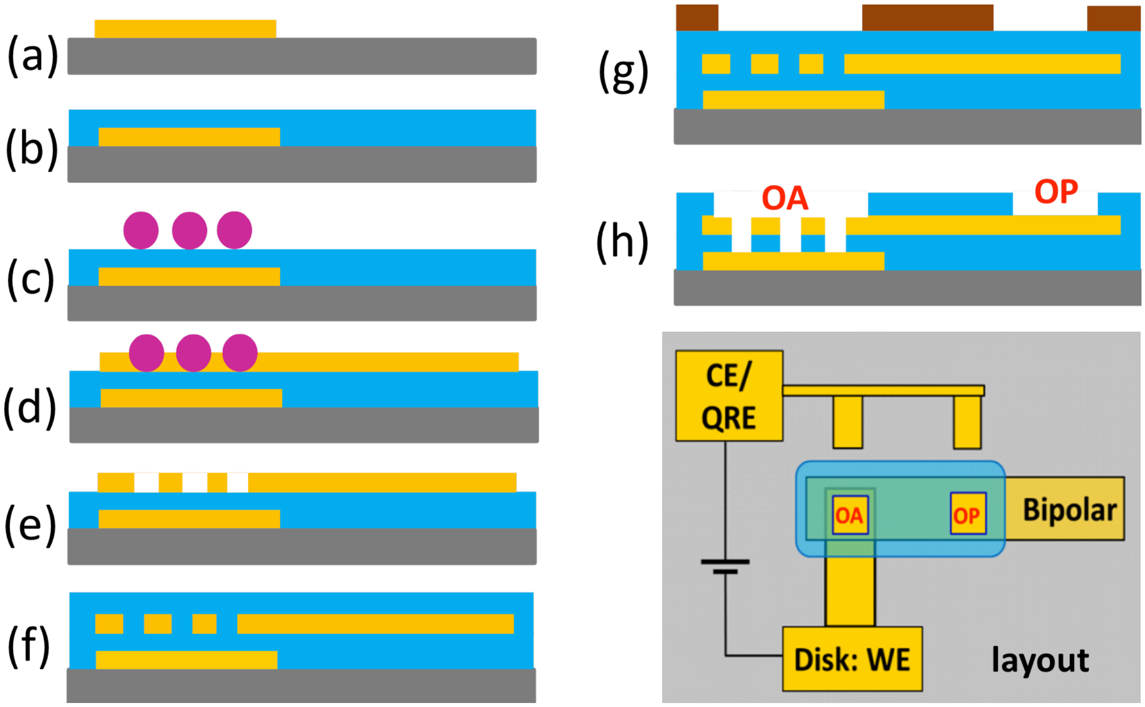

Figure S1. Schematic illustration of the fabrication procedure and the layout for the BRDE array. The colors are used to represent different layers: grey (glass), yellow (Au), blue (SiNx), purple (polystyrene sphere), and brown (photoresist). OA and OP are the open areas defined in the main text.

2. CV results showing dependence of the SIRC effect on the size of the exposed area on the bipolar electrode.

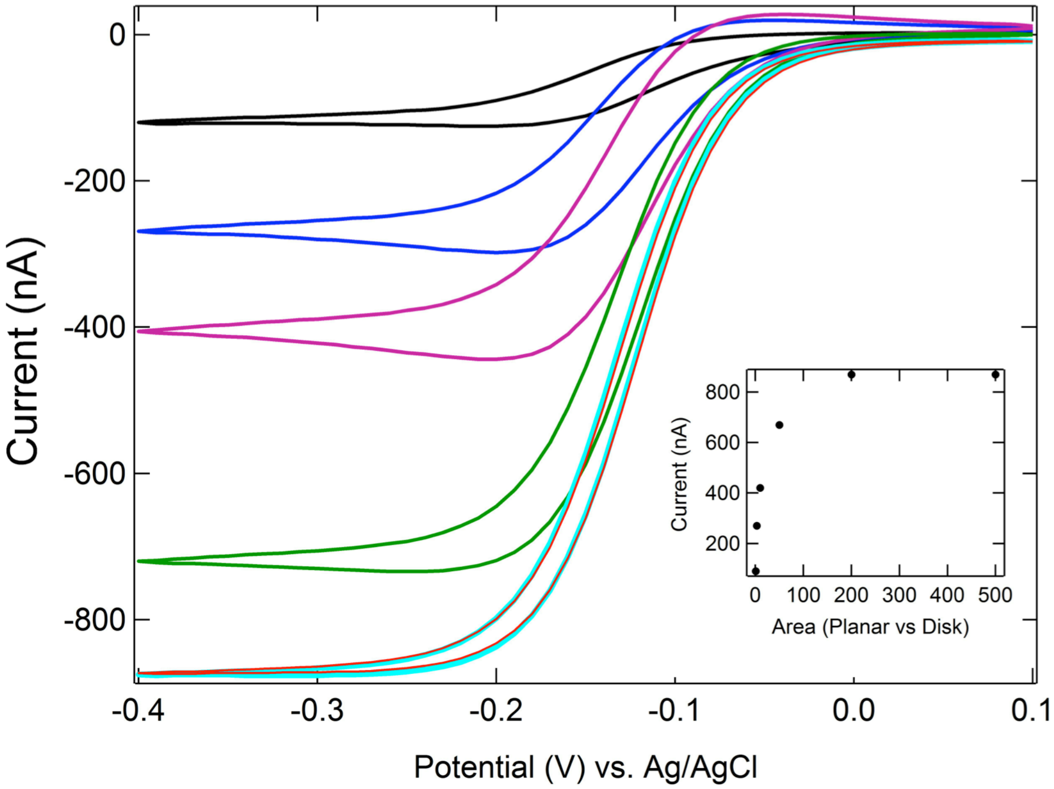

Figure S2. Cyclic voltammograms of 1 mM $Ru(NH_3)_6^{3+}$ on the BRDE array with both OA (100 µm x 100 µm) and OP (varying area) in contact with redox-active species. The ratio of the area of the remote bipolar (OP) to the disk (OA) electrode is 1 (black), 3 (blue), 10 (purple), 50 (green), 200 (cyan), and 500 (red), respectively. (*Inset*) limiting current as a function of bipolar area *vs*. disk area.

3. Optical image of ECL measurement showing the change of the ECL intensities on the disk (OA) and bipolar electrode (OP) with concentration of TPA

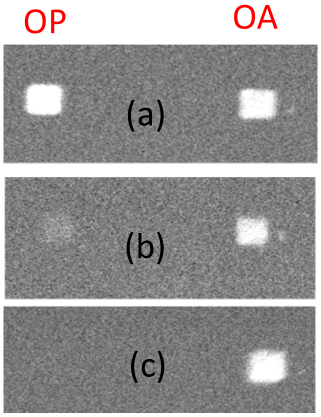

Figure S3. Optical image of ECL measurements on the BRDE array in 5 mM $Ru(bpy)_3^{2+}$ and 20 mM (a), 50 mM (b), and 100 mM (c) TPA.

4. ECL measurements on microdisk electrode showing the change of ECL intensities with the concentration of TPA

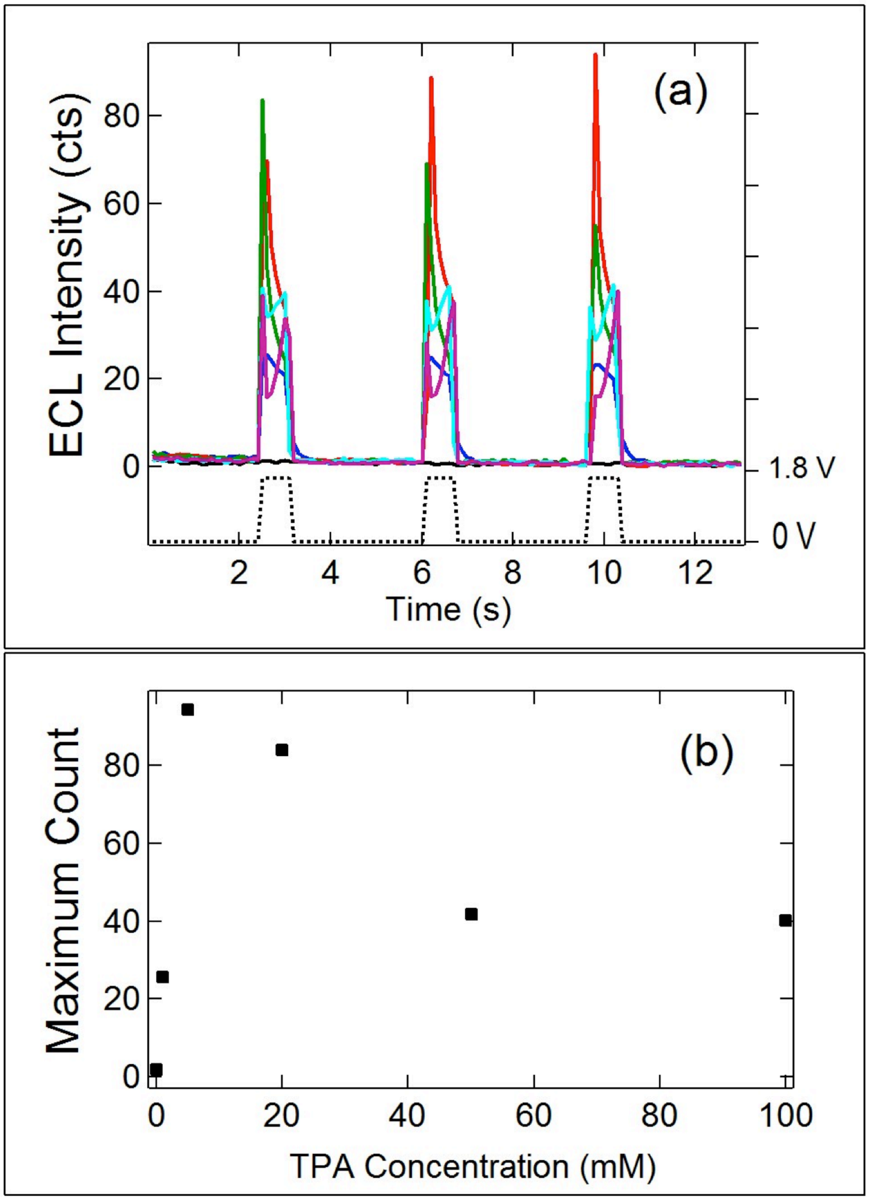

Figure S4. (a) (*Top*) Potential modulated ECL on a microdisk electrode in 5 mM $Ru(bpy)_3^{2+}$ and 0 mM (black), 1 mM (blue), 5 mM (red), 20 mM (green), 50 mM (cyan) and 100 mM (purple) TPA; (*Bottom*) Applied potential *vs.* QRE as a function of time. (b) The maximum ECL intensity as a function of TPA concentration

5. CV results showing dependence of the SIRC effect on the concentration of the analyte in the remote portion (OP) of the bipolar electrode.

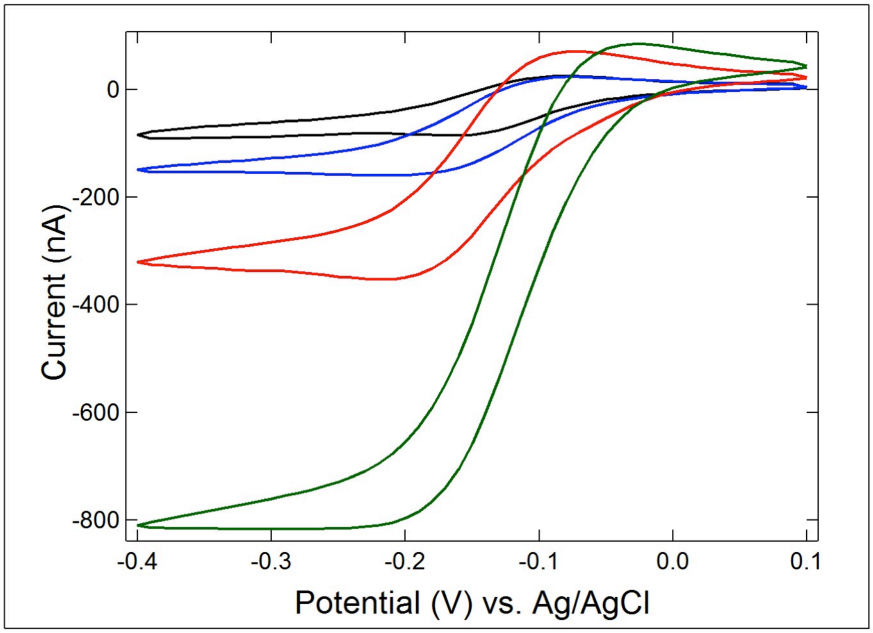

Figure S5. Cyclic voltammograms on the BRDE array with area OA in contact with 1 mM $Ru(NH_3)_6^{3+}$ while area OP is in contact with 0 mM (black), 1 mM (blue), 10 mM (red), and 100 mM (green) $Ru(NH_3)_6^{3+}$ solution, respectively.

References

(1) Ma, C.; Contento, N. M.; Gibson, L. R.; Bohn, P. W. *ACS Nano* **2013**, *7*, 5483.

(2) Li, H.; Wu, N. *Nanotechnology* **2008**, *19*, 275301/1.
